# Obstructive Sleep Apnea: A Contributing Factor in Gout

**DOI:** 10.7759/cureus.52115

**Published:** 2024-01-11

**Authors:** Pushti Khandwala, Devashish Desai, Mitali Sen

**Affiliations:** 1 Rheumatology, Einstein Medical Center Philadelphia, Philadelphia, USA; 2 Hematology and Oncology, State University of New York Upstate Medical University, Syracuse, USA; 3 Rheumatology, Birmingham School of Medicine, University of Alabama, Birmingham, USA

**Keywords:** clinical rheumatology, risk factor analysis, big data analysis, obstructive sleep apnea (osa), gout

## Abstract

Introduction: Obstructive sleep apnea (OSA) is a comorbidity, which has shared risk factors with gout as well as causes pathophysiological mechanisms causing hyperuricemia. The relationship remains contentious.

Methods: TrinetX, a global federated research network that provides a dataset of electronic medical records from different healthcare organizations (HCOs). We utilized this network to query patients who had a BMI greater than 30 and then two subgroups were made based on the presence or absence of OSA. Furthermore, propensity score matching (PSM) was carried out to match age, sex, race, chronic kidney disease (CKD), heart failure, and the use of diuretics. Compare outcome analytic function was utilized to map the co-relation with Gout.

Results: A total of 3541566 patients who had a BMI >30 were identified, out of which 817638 (23.09%) patients had OSA. 7.19% of patients with OSA had gout while 2.84% without OSA had gout (p<0.0001). The odds of having gout are 2.65 times higher in patients with OSA than patients without OSA (hazard ratio is 2.393, 95% confidence interval (CI) 2.367-2.419, p<0.0001). After PSM, both the groups of obese patients with and without International Classification of Diseases, 10th Revision (ICD-10) diagnosis of OSA included 801526 patients, within which 6.93% of patients with OSA had gout while 4.63% of patients without OSA had gout (p<0.0001). The odds ratio was 1.533 (95% CI 1.512-1.554, p<0.0001) and the hazard ratio was 1.404 (95% CI 1.386-1.423).

Conclusion: Our study demonstrated that there is a strong correlation between gout and OSA. Chronic hypoxia-induced hyperuricemia is the most widespread explanation. OSA is a treatable condition with timely diagnosis and proper treatment. Prospective cohort studies are required to further test the strength of the relationship between OSA and gout.

## Introduction

Gout is the most common inflammatory arthritis globally, with an incidence of 7.44 million cases and a prevalence of 41.22 million cases worldwide in 2017, causing 1.28 million disability-adjusted life years (DALYs) [1]. Gout flares are episodes of recurring acute inflammation precipitated from the deposition of monosodium urate crystals in the joints and tendons due to persistently elevated serum uric acid levels. The prevalence of gout in the United States of America is high, per the 2015-2016 National Health and Nutrition Examination Survey (NHANES) was 3.9% [2]. It is associated with common comorbidities like metabolic syndrome, cardiovascular disease, insulin resistance, obesity, and hypertension [3,4]. Obstructive sleep apnea (OSA) is another widely prevalent disease affecting 2-4% of the global population and has similar shared risk factors with gout [5]. It affects at least 25 million Americans, with the annual economic burden of undiagnosed sleep apnea being about $150 billion [6].

There have been various hypotheses placed forward that can contribute to the increased association of gout and OSA. Sleep apnea-induced hypoxemia can cause an increase in purine concentrations by increased degradation of adenosine triphosphate (ATP), eventually increasing uric acid levels [7]. Acidosis caused by hypercapnia in OSA can facilitate monosodium urate monohydrate (MSU) nucleation [8]. Lactic acid generated during neutrophilic phagocytosis of already deposited crystals in synovium causes further lowering of the pH, which further promotes local impetus for crystal formation [9]. Moreover, lactic acid generated during hypoxic episodes in OSA can lead to higher renal reabsorption of uric acid, further increasing its levels [10].

There have been studies, mainly in the UK, which have shown a 1.5 times increased risk of gout in patients with OSA [11,12]. On the contrary, a study by Durme et al. shows that the association disappears after statistical adjustment for BMI, renal function, heart failure, and use of diuretics [13]. Thus, it becomes vital to contribute to the existing literature to further investigate this relationship.

This article was presented in the format of an oral presentation at (The European Alliance of Associations for Rheumatology) EULAR 2023 in Milan, Italy on June 2, 2023.

## Materials and methods

This is a retrospective, non-interventional cohort study. The data was obtained from TrinetX, which is a novel, global federated research network integrating a wide array of data collected from electronic medical records continuously, from over 130 major healthcare systems worldwide, including academic medical centers, specialty hospitals, specialty physician practices, and community hospitals, collectively referred to as healthcare organizations (HCOs).

Study population

We identified all patients with a primary diagnosis of "BMI of more than or equal to 30” by querying the database using the International Classification of Disease - Clinical Modification, 10th Revision (ICD-10 CM) code “Z68.3”. The study population was divided into two groups, one with OSA and one without. The cohorts then were studied for the prevalence of gout. All the ICD codes used in the study are mentioned in Table 1.

**Table 1:** ICD codes used in the study ICD, International Classification of Diseases; CKD, chronic kidney disease

ICD-10 code	Description
Z68.3	BMI ≥30
G47.33	Obstructive sleep apnea
N18	CKD
I50	Heart failure
Pharmaceutical catalog	
VA CV700	Diuretics

Study analysis

The analysis was performed using TrinetX. A two-tailed alpha of <0.05 was required for statistical significance. Baseline characteristics of participants were summarized using descriptive statistics. Continuous data was presented as means and standard deviation and compared using a t-test, while categorical data was presented as percentages and proportions and compared using a chi-square test. We evaluated the prevalence of gout in patients with and without a history of OSA. After the tests on raw data, propensity score matching (PSM) was performed to reduce selection bias and the effects of confounding variables by balancing the covariates between the two groups.

## Results

A total of 3541566 patients were identified who had a BMI ≥30, out of which 817638 (23.09%) had OSA. Table 2 shows the demographics of the study population. Patients in the OSA group were older compared to patients in the non-OSA group (59.8±14.3 years vs. 54±17.2 years, p<0.0001). 50% (n=408819) of the patients with OSA were females vs. 65% (n=1770553) in the group without OSA (p<0.0001). Caucasians were the predominant race followed by African Americans (p<0.0001). Unknown race and Asians had a higher prevalence in the non-OSA group than in the OSA group. Heart failure, CKD, and the use of diuretics were also more prevalent in the OSA group.

**Table 2:** Demographics CKD, chronic kidney disease

Variables		OSA		P-value
		Yes	No	
Age (means)		59.8±14.3	54±17.2	<0.0001
Gender (%)	Females	50	65	<0.0001
	Males	50	35	
Race (%)	White	70.08	67.33	<0.0001
	African Americans	18.1	18.56	
	Asians	0.75	1.17	
	American Indians or Alaska Native	0.37	0.38	
	Native Hawaiian and Pacific Islanders	0.14	0.18	
	Unknown	10.56	12.38	
Heart failure (%)		13	3.31	<0.0001
CKD (%)		10.88	4.18	<0.0001
Use of diuretics (%)		36.96	16.76	<0.0001

It was seen that the prevalence of gout was 7.19% (n=58788) in the OSA group while it was only 2.84% (n=77360) in the non-OSA group (p<0.0001). Patients with OSA had 2.65 times higher odds of having gout than those without OSA (OR 2.65, 95% CL 2.576-2.634, p<0.0001). The hazard ratio for developing gout was 2.393 in OSA patients compared to non-OSA patients (HR 2.393, 95% CL 2.367-2.419, p<0.0001). 

PSM was done, where age, race, gender, CKD, heart failure, and use of diuretics were matched. It was seen that the prevalence of gout was still higher in the OSA group than in the non-OSA group (6.93% (n=55,546) vs 4.63% (n=37,111), p <0.0001). Patients with OSA had 1.533 times higher odds of having gout than those without OSA (OR 1.533, 95% CI 1.512 - 1.554, p<0.0001). The hazard ratio for developing gout was 1.404 in OSA patients compared to non-OSA patients (HR 1.404, 95% CI 1.386-1.423, p<0.0001).

## Discussion

The novelty of this study is that we utilized electronically recorded data from over 130 major healthcare systems worldwide through TrinetX, hence, getting a global analysis of the association between gout and OSA. In our study, it was seen that the population with OSA was elderly, predominantly males, and had a higher prevalence of heart failure, CKD, and use of diuretics. Gout is associated with many of the same comorbidities, which are prevalent in OSA. Elderly and males are known to have an increased prevalence of gout [1]. Heart failure has been associated with gout for a long time. There have been studies that show that gout (hyperuricemia) is a modifiable risk factor for heart failure and treating it with the use of uric acid-lowering agents has shown favorable effects on myocardial function [14,15]. Vice versa, the medications used in heart failure like diuretics, beta-blockers, angiotensin-converting enzyme inhibitors, and non-losartan angiotensin II receptor blockers have been shown to cause elevation of uric acid, leading to gout [16-18]. As 70% of uric acid is excreted from the kidneys, hyperuricemia occurs when renal function deteriorates [19]. Matching for the comorbidities strengthens the results of the study by allowing a direct association.

Our study demonstrated that there is a higher odds of having gout in patients with OSA, which remains statistically significantly high after PSM for age, race, gender, CKD, heart failure, and use of diuretics. Patients with OSA had 2.65 times higher odds of having gout than those without OSA, and after matching, the odds ratio was 1.533. This contrasts with the study by Durme et al. in 2020, which showed that the association between gout and OSA had disappeared after adequate statistical adjustment for BMI, renal function, heart failure, and recent use of diuretics [14]. Regardless, the drop in odds ratio does shine a light that OSA and gout have many confounding or associated comorbidities. Similarly, the hazard ratio was 2.393 before matching, which dropped to 1.404 after matching.

Study limitation

Being a retrospective observational study, we can only determine the association between the conditions; to get accurate causality, prospective studies need to be done. Also, the limitations are associated with administrative errors in coding.

## Conclusions

In conclusion, our study showed that there is a relationship between Gout and OSA, which remains significant after adjusting for demographics, heart failure, CKD as well as the use of diuretics. OSA is a modifiable risk factor and shares similar comorbidities with gout. Though prospective studies are required for determining causality, medical practitioners should be vigilant about OSA being a risk factor in the development of gout. There is also a need for studies evaluating the role of OSA treatment in primary prevention of gout or secondary prevention of gout flares. 
